# Parent and Clinician Preferences for an Asthma App to Promote Adolescent Self-Management: A Formative Study

**DOI:** 10.2196/resprot.5932

**Published:** 2016-12-06

**Authors:** Lorie L Geryk, Courtney A Roberts, Adam J Sage, Tamera Coyne-Beasley, Betsy L Sleath, Delesha M Carpenter

**Affiliations:** ^1^Division of Pharmaceutical Outcomes and PolicyEshelman School of PharmacyUniversity of North CarolinaChapel Hill, NCUnited States; ^2^Division of General Pediatrics and Adolescent MedicineSchool of MedicineUniversity of North CarolinaChapel Hill, NCUnited States

**Keywords:** asthma, self-management, social support, mHealth, mobile apps, adolescents, parents, clinicians

## Abstract

**Background:**

Most youth asthma apps are not designed with parent and clinician use in mind, and rarely is the app development process informed by parent or clinician input.

**Objective:**

This study was conducted to generate formative data on the use, attitudes, and preferences for asthma mHealth app features among parents and clinicians, the important stakeholders who support adolescents with asthma and promote adolescent self-management skills.

**Methods:**

We conducted a mixed-methods study from 2013 to 2014 employing a user-centered design philosophy to acquire feedback from a convenience sample of 20 parents and 6 clinicians. Participants were given an iPod Touch and asked to evaluate 10 features on 2 existing asthma apps. Participant experiences using the apps were collected from questionnaires and a thematic analysis of audio-recorded and transcribed (verbatim) interviews using MAXQDA. Descriptive statistics were calculated to characterize the study sample and app feature feedback. Independent samples *t* tests were performed to compare parent and clinician ratings of app feature usefulness (ratings: 1=not at all useful to 5=very useful).

**Results:**

All parents were female (n=20), 45% were black, 20% had an income ≥US $50,000, and 45% had a bachelor’s degree or higher education. The clinician sample included 2 nurses and 4 physicians with a mean practice time of 13 years. Three main themes provided an understanding of how participants perceived their roles and use of asthma app features to support adolescent asthma self-management: monitoring and supervision, education, and communication/information sharing. Parents rated the doctor report feature highest, and clinicians rated the doctor appointment reminder highest of all evaluated app features on usefulness. The peak flow monitoring feature was the lowest ranked feature by both parents and clinicians. Parents reported higher usefulness for the doctor report (t_(10)_=2.7, *P*<.02), diary (t_(10)_=2.7, *P*<.03), and self-check quiz (t_(14)_=2.5, *P*<.02) features than clinicians. Specific participant suggestions for app enhancements (eg, a tutorial showing correct inhaler use, refill reminders, pop-up messages tied to a medication log, evidence-based management steps) were also provided.

**Conclusions:**

Parent and clinician evaluations and recommendations can play an important role in the development of an asthma app designed to help support youth asthma management. Two-way asthma care communication between families and clinicians and components involving families and clinicians that support adolescent self-management should be incorporated into adolescent asthma apps.

## Introduction

Youth with asthma often have complex self-management regimens that include responding to changes in symptoms, taking multiple medications, dealing with doctor consultations, and adjusting to treatment changes [1]. The asthma care process (eg, developing and implementing an asthma management plan) should involve collaboration between the clinician, the patient, and his/her parents [2,3]. Self-management support should be provided to families in between medical visits, using a style that reflects individual needs and with an understanding that youth with asthma generally take on greater self-care autonomy with age [1,4]. Better clinician and caregiver involvement and support for self-management is known to improve asthma control among children and adolescents [5-8], and, increasingly, digital technologies (eg, Internet and mHealth apps) are being used to deliver that support [9]. While asthma apps offer a promising method for providing self-management support [10], little is known about how parents and clinicians would prefer to use an asthma self-management app to promote adolescent self-management.

Internet interventions designed to facilitate adolescent asthma self-management often take a fragmentary approach by addressing information, decision support, or social support individually, and not as part of a comprehensive asthma management strategy [11]. In a review, Morrison et al [9] found that most digital asthma self-management interventions incorporated an average of 4 of the following common features: asthma self-care education, asthma action plan, self-monitoring, interactive/receiving immediate feedback from the device, alerts from device, alerts from health professionals, and games. Interventions rarely possessed features that encouraged two-way asthma care communication between families and clinicians or features to help families and clinicians facilitate adolescent self-management. In fact, in 2013, only 1% of available apps (3/209) targeted both children and their caregivers [12]. As of 2015, in a review of asthma-related apps by Wu et al [13], only 8% (9/209) of apps were child-focused, with only 2 including adolescents and neither having components for parents or clinicians.

Few studies have sought to assess clinician and parent perspectives on adolescent asthma self-management apps [14-16]. In a pilot study, Haze et al [14] found that nurses believed the use of smartphone apps improved their ability to contact adolescents and increased the accuracy of clinical assessment. In another study, parents suggested medication and refill reminders and alerts (eg, teen to call the doctor when peak flow readings are concerning) when asked what they would like to see in a digital asthma tool for teens with asthma [15]. Another study found that clinicians believed apps could assist patients in developing self-management skills and serve as a two-way channel for sharing accurate and credible information [16].

This study adds to the existing literature because it simultaneously considers clinician and parent perspectives related to a wider-ranging array of app self-management features (eg, diary, chart, self-check quiz, reminder alerts) than have previously been considered. This study is part of a larger exploratory pilot project proposing to develop a comprehensive mHealth adolescent-centered asthma self-management app. The overall goals of the parent study are to design and test a theory-based adolescent centered mHealth app and to examine adolescent patient satisfaction with and feasibility of the app prototype. The purpose of this study was to gather data relevant to designing future adolescent asthma self-management apps by having parents and clinicians evaluate features from 2 existing asthma apps. Specifically the goals were to obtain (1) general app and specific feature impressions, (2) suggestions for modifications and new features, and (3) to identify ways participants would like to be included in app use to more effectively support adolescent asthma self-management behaviors.

## Methods

### Recruitment

For this formative study, we gathered ideas for future adolescent asthma self-management app features by leveraging the opinions and feedback of a convenience sample of 20 caregivers and 6 clinicians (2 nurses and 4 physicians). Participants were recruited from 2 pediatric practices located in an urban area of North Carolina between 2013 and 2014. This study is part of a larger study that explored adolescent feedback and theoretical pathways through which asthma app features can promote adolescent self-management [17,18].

### Study Procedures

A designated clinic liaison used the eHealth record to identify adolescents with asthma and informed parents about the study prior to the adolescent’s regularly scheduled clinic appointment. Upon arriving for the adolescent’s appointment, interested parents were introduced to the study research assistant (RA). Parents were eligible if they (1) were at least 18 years of age, (2) could read and understand English, and (3) were the biological parent or legal guardian of an adolescent (12-17 years of age) with persistent asthma, which was defined as experiencing asthma-related daytime symptoms more than twice a week, asthma-related nighttime symptoms more than twice a month, or receiving one or more long-term control therapies for asthma [19,20].

After providing consent and prior to the adolescent’s appointment, parents completed a brief questionnaire that assessed demographics and technology use. After the appointment, the RA gave the parent an iPod Touch that contained 2 asthma self-management apps; one app targeted adults and one targeted children. The 2 apps were purposely selected from those that were available on the iOS platform in late 2012 because they had the combined characteristics of being user-friendly and having multiple self-management features. At the time, no app was targeted to adolescents or had components designed to incorporate caregiver or clinician use. The RA demonstrated how to use the 2 apps and then allowed parents to explore the apps on their own for approximately 10 minutes. Parents were then asked to explore and use features of both apps over the course of 1 week and completed a 20- to 30-minute semistructured telephone interview that assessed their experience using the apps, perceived usefulness of app features, how app features could be improved, and suggestions for new features.

Three clinicians were recruited from each pediatric practice. Clinicians were eligible for a 30-minute in-person interview if they provided health care for adolescents with asthma. After providing consent and prior to being given the iPod Touch, clinicians answered demographic and technology use surveys. The RA then demonstrated how to use the 2 apps and then allowed clinicians to explore the apps on their own for approximately 10 minutes prior to completing a semistructured interview to obtain feedback on app use and usefulness of specific app features. Clinicians were not asked to evaluate the apps for a longer period due to professional time-constraints.

All interviews were digitally audio recorded. Parents and clinicians were allowed to keep their iPod Touches as incentives. The study was reviewed and approved by the University of North Carolina Institutional Review Board institutional review board and was conducted in accordance with the tenets of the Declaration of Helsinki.

### Measures

#### Demographic and Clinical Characteristics

Parents and clinicians reported their gender, age, and ethnicity (Hispanic, Latino or Spanish origin). Race was measured as a categorical variable (White, Black or African American, American Indian or Alaskan Native, Asian, Native Hawaiian and Other Pacific Islander, or Other) and, for descriptive purposes, was recoded into the following categories: White, Black, and Other. Parents reported their highest level of education (less than high school; high school graduate or general education development; some college, no degree; associate’s degree; bachelor’s degree; more than bachelor’s degree) and annual household income (<US $25,000, US $25,000 to US $34,999, US $35,000 to US $49,999, US $50,000 to US $74,999, and ≥US $75,000). Clinicians reported occupation and years in practice.

#### Technology Use

Parents and clinicians answered the following close-ended questions to report: owned a cell phone (yes/no), what type of cell phone they used (iPhone, android, other “basic”), owned another Internet-capable device (ie, computer, tablet, or iPad) (yes/no). Using a yes/no response format, we also asked parents and clinicians if they had (1) ever downloaded an app to a cell phone, tablet, or other handheld device, (2) ever downloaded a health-related app, (3) ever paid to download an app, or (4) ever avoided downloading an app due to privacy concerns. Parents were also asked about their interest in using an app to watch videos of correct inhaler technique, to take asthma quizzes, and to send information about the child’s health to the doctor.

#### App Use Questions and App Feature Usefulness Ratings

Parents and clinicians were asked, “[w]hat did you think of (feature name here)?” The following 10 features were asked about: diary, chart, self-check quiz, allergies and emergency plan, doctor appointment reminder, medication reminder, asthma triggers, doctor report, school form, and peak flow. Parents and clinicians were asked additional open-ended questions about how the app or specific features could be changed and improved upon to make them better and if they had any ideas for new app features that they would like to see included in an app. Parents were asked which features they used the most and why, and which features they used the least and why. Parents and clinicians were also asked to rate the 10 app features on a scale from 1 to 5 (1=not at all useful to 5=very useful).

Parents were asked the following four questions: (1) After using the apps would you say that it helped you to better monitor your child’s asthma? (2) Do you believe that using the apps helped you control your child’s asthma better than you have in the past? (3) Do you feel more involved in managing your child’s care after using the apps? (4) Would you say using an app helped you follow the doctor’s advice better? Clinicians were asked if they thought that “using an app would help parents and adolescents…” (1) better monitor their child's/their asthma, (2) control their child's/their asthma better than they would without using an app, (3) feel more involved in asthma management, and (4) follow their advice better.

### Data Analysis

Quantitative statistical analyses were conducted using IBM SPSS Version 21. Each interview was transcribed verbatim, de-identified, and analyzed thematically using MAXQDA software. Descriptive statistics were calculated to characterize the study sample. Independent samples *t* tests were performed to compare parent and clinician ratings of app feature usefulness. For qualitative data, 3 research team members engaged in an iterative process of reading and rereading the initial transcripts [21] in order to identify relevant themes and create a detailed codebook with code definitions and example quotations that was applied to all transcripts [22]. The codebook included codes for app features, facilitators and barriers to app use, asthma self-management (eg, medication management), and information related to improving app features. These codes were then placed within 3 major theme categories related to how clinicians and parents could use apps to support adolescent self-management: monitoring/supervision, communication/information sharing, and education. Monitoring and supervision related to using apps to oversee and be involved in various aspects of the adolescents’ asthma care and management. Communication/information sharing related to using apps to give and receive information and discuss information effectively with social network members. Education concerned use of the app as a teaching and support tool to encourage learning about asthma. After the codebook was finalized, a primary coder coded all interview transcripts and a secondary coder coded 10% of the transcripts; inter-rater reliability was good (k=0.85).

## Results

### Sample Characteristics and Technology Use

Table 1 presents the demographic characteristics of parents (n=20) who participated in the study. The clinician sample included 2 nurses and 4 physicians with a mean practice time of 13 years. Clinicians were 83% female (5/6), 67% white (4/6), 17% Hispanic (1/6), and 40-years old on average. Table 2 shows reported technology use of both parents and clinicians. Of parents 50% (10/20) reported often seeking out Web-based medical information about asthma, but 80% (16/20) had never used an asthma app before the study. Four parents did not complete their 1-week follow-up telephone interview, and these individuals were not demographically different from those who completed the study.

**Table 1:** Parent demographics.

Demographics		(n)/mean	%/(standard deviation)
**Gender**			
	Male	(0)	0
	Female	(20)	100
Age		44	(±8.4)
**Highest level of education completed**			
	Less than high school	(2)	10
	High school graduate or GED	(1)	5
	Some college, no degree	(6)	30
	Associate's degree	(2)	10
	Bachelor's degree	(5)	25
	More than bachelor's degree	(4)	20
**Total household income before taxes during (last 12 months)**			
	Less than US $25,000	(7)	35
	US $25,000-$34,999	(3)	15
	US $35,000-$49,999	(2)	10
	US $50,000-$74,999	(2)	10
	US $75,000-$99,999	(1)	5
	US $100,000-$149,999	(1)	5
	US $150,000 or more	(2)	10
	Refused	(2)	10
**Race**			
	White	(9)	45
	Black	(9)	45
	Other (all options selected)	(1)	5
	Missing	(1)	5
**Ethnicity**			
	Hispanic/Latino/Spanish	(4)	20

### Overall App Use Impressions

#### Monitoring and Supervision

Overall, parental opinions for using the apps to monitor and supervise their child's asthma were positive. One parent of a 14-year-old girl stated, “[i]t’s a very good digital record-keeper.” Clinicians generally had positive things to say about the apps as a self-management tool to help parents and adolescents including the following: “hands-on” and provides a “more interactive or fun way to check on their asthma.”

Of parents, 80% (16/20) said that using an app helped them better monitor their child's asthma, and 40% (8/20) believed that using an app helped them control their child's asthma better than they had in the past. Fifty percent (10/20) of parents felt more involved in managing their child's asthma after using an app, and half felt that using an app helped them follow their child's doctor's advice better.

Of clinicians, 50% (3/6) thought that using an app would help parents and adolescents better monitor their child's/their asthma, and 83% (5/6) believed that using an asthma app would help parents/adolescents control their child's/their asthma better than they would without using an app. All clinicians (6/6, 100%) thought that parents and adolescents would feel more involved in managing asthma by using an app, and 83% (5/6) thought that asthma apps would help parents and adolescents follow their advice better.

#### Communication/Information Sharing

Overall, parent and clinician opinions of using the apps for communication/information sharing purposes were positive. Of parents, 80% (16/20) said that they would use an app to send information about their child to their child's doctor. One parent pointed out the benefit of having asthma information in an app form, over paper, so that the information is in one place to be pulled up and easily shared. One clinician stated,

As a nurse, I would love to communicate with my patient through it. It's a better tracking device. Kids always have their phone in their hand.

Clinicians felt that use of the app could lead to a better medical appointment both in terms of efficiency, patient-centered care, and decision making. Multiple clinicians expressed data security concerns (eg, insecure email) or differed in their preference for information delivery method; some preferred email, some preferred hand-delivery or hard copy print-outs, and others preferred the phone.

**Table 2:** Parent and clinician technology use.

Technology use		Parents	Clinicians
		n (%)	n (%)
**Do you own a cell phone?**			
	Yes	18 (90)	6 (100)
	Missing	2 (10)	-
**Type of phone**			
	iPhone	9 (45)	6 (100)
	Android	8 (40)	-
	Other “basic”	1 (5)	-
**Do you own any other Internet-capable device?^a^**			
	Computer	19 (95)	6 (100)
	Tablet	7 (35)	5 (83)
	iPad	4 (20)	4 (67)
	Other (laptop)	1 (5)	1 (17)
**Have you downloaded an app to a cell phone, tablet, or other handheld device?**			
	Yes	19 (95)	6 (100)
	No	1 (5)	-
**Have you ever downloaded a health-related app to a cell phone, tablet, or other handheld device?**			
	Yes	10 (50)	5 (83)
	No	10 (50)	1 (17)
**Have you ever paid to download an app?**			
	Yes	6 (30)	5 (83)
	No	14 (70)	1 (17)
**Have you ever avoided downloading an app due to concerns about sharing your personal information?**			
	Yes	11 (55)	2 (33)
	No	9 (45)	4 (67)

^a^Participants could report owning more than one device.

#### Education

There were 75% of parents (15/20) who said that they would use an app to watch videos of correct inhaler technique, and 80% (16/20) would take asthma quizzes if they were part of the app. One clinician thought that the use of an app would serve to reinforce important concepts that they heard in the doctor’s office such as why daily controller medication is useful. Parents and clinicians thought an app would help reduce confusion about the difference between a rescue medication and maintenance medication. One clinician had this to say:

I think most of them engage in devices like this for entertainment, right? And so you want to have something that provides them an educational opportunity, um, but also something that they – they won’t get bored with.

**Table 3:** App features and their relationship to self-management support.

App feature (major theme category)	Feature description	Parent and clinician example quotes
Diary (monitoring/supervision)	A place to review past asthma data entered for each day, including peak flows, selected symptoms and triggers (adult app).	Related theme: monitoring/supervision *Parent: I like the diary part because they can – I can see the days that they dipped, the days that were good. And then I can go back and see, okay, well, she was on a bad day on this day[….] You know, just kind of me being able to really see, you know, how she’s doing, when, the how, when and where of how functions are actually doing.*
Chart (monitoring/supervision)	At-a-glance, graphical view of peak flow meter readings (adult app).	Related theme: monitoring/supervision *Clinician: So if you’re utilizing peak flow, I think that’s good, um, and if you don’t have a sense of where you typically run, having this long, longer, um, snapshot of what. your peak flows typically are could be helpful.*
Self-check quiz (monitoring/supervision, education)	Seven questions that assess asthma control and output a numerical score telling the user how well-controlled their asthma is and to discuss the results of quiz with caregivers and physician (child app).	Related theme: education *Parent: I think it, it’s a good teaching tool for him.*
Allergies and emergency plan (monitoring/supervision, education, communication/ information sharing)	A place to document emergency contact and doctor’s phone number, best and recent peak flow records, allergy medications and dosage, and an allergic reaction plan (child app).	Related theme: education *Clinician: Um–, we give patients an asthma action plan, but if that’s sitting at home on the refrigerator and they’re out at the grocery store and they don’t remember what they’re supposed to do having something that’s right there with them...I think, um, would be really useful.*
Doctor appointment reminder (monitoring/supervision)	Enter doctor's appointments in icalendar (ical) to receive a text reminder (child app).	Related theme: monitoring/supervision *Parent: That’s good because – so I don’t forget. I don‘t forget the, the appointment, that – that’s good.*
Medication reminder (monitoring/supervision)	Allows user to enter daily and emergency medications, input times they take their medications in ical, and then request a daily text reminding them to take their medication. Alerts user when it is time to take their medications (adult app). Allows users to drag and drop the medication into the open mouth of a monster to verify that medication has been taken (child app).	Related theme: monitoring/supervision *Clinician: Um, I think tracking medications is critical, and reminding –having a feature within the app that you can remind patients to take their medications and also track when they actually took them. So, you know, like you – where they – the app feeds the med to the monster’s mouth I think that’s really useful.*
Asthma triggers (monitoring/supervision, education, communication/information sharing)	A place for selecting triggers from a preexisting list as well as a place to add custom asthma triggers [adult app].	Related theme: monitoring/supervision *Parent: It keeps the triggers of what triggers her asthma in front of us. Um, so we know what we’re looking for and how she might turn out that day just because of the weather or because she had to do certain exercises, um, so that reminds us to keep her rescue inhaler with her during, um, those different times.*
Doctor report (monitoring/supervision, communication/information sharing)	Allows patient to share asthma record with care team by sending an email to them through the app to view record (adult app).	Related theme: communication/information sharing *Clinician: If I had a patient that could send me information on if they were doing...peak flow, what it’s been and what your symptoms have been in the last two weeks, like...that would be really easy for me to, um, say, “You need a visit, we need to up your control or we need to,- whatever.*
School form (communication/information sharing)	Physician approval forms that allow students to possess and use an inhaler in school. Form can be emailed from the app to the physician. Physician can print form or fill it out electronically and email to child's school (child app).	Related theme: communication/information sharing *Clinician: That saves us some [time], and plus they have a copy and it goes straight to the school. And everyone is on the same page.*
Peak flow (Monitoring/supervision)	A place where one can enter their daily peak flow measurements. The information can be relayed to chart and visualized in graphical format (adult app).	Related theme: monitoring/supervision *Parent: That part’s really not useful to us…cause she doesn’t have to do peak flows on -- on a regular basis, so, um, we only do those when she’s had an attack and then they want us to keep track of them for a week and then she doesn’t have to do them anymore.*

### App Features

Table 3 provides a list of the app features that parents and clinicians evaluated. Feature descriptions are also provided as well as example quotes associated with major theme categories (monitoring/supervision, education, communication/information sharing) for each feature. Monitoring and supervision was discussed in relationship with 9 of 10 app features. Communication/information sharing was discussed in relationship with 4 of 10 app features. Education was discussed in relationship with 3 of 10 app features.

#### Perceived Usefulness of App Features

**Figure 1:**
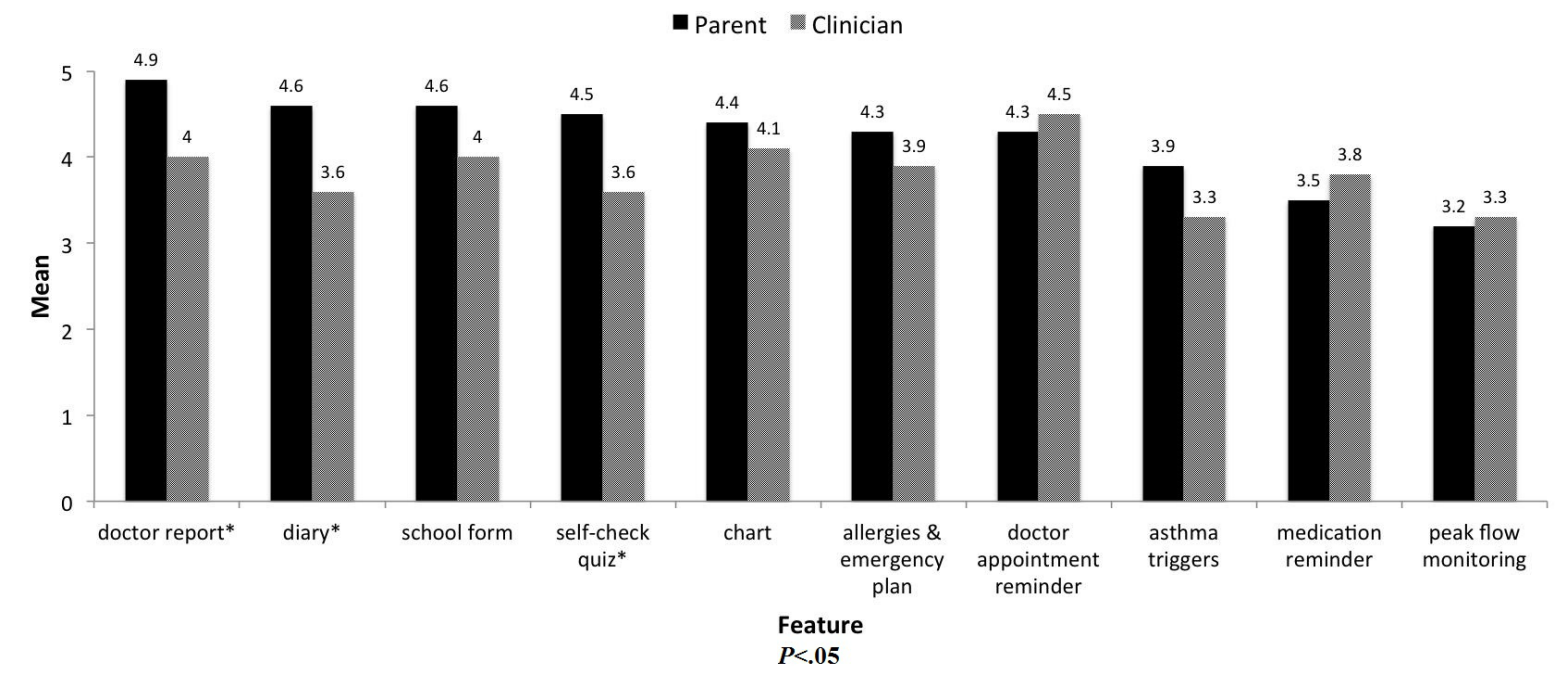
Parent and clinician app feature usefulness rankings (note: mean feature usefulness ratings on a scale of one to five; 1=not at all useful to 5=very useful).

Figure 1 presents the usefulness of app features as ranked by parents (ordered highest to lowest) and clinicians. Parents rated the doctor report highest (4.9/5) and clinicians rated the doctor appointment reminder highest (4.5/5) of all app features on usefulness. The peak flow feature was the lowest ranked feature by both parents (3.2/5) and clinicians (3.3/5). Parents reported higher usefulness for the doctor report (t_(10)_=2.7, *P*<.02), diary (t_(10)_=2.7, *P*<.03), and self-check quiz (t_(14)_=2.5, *P*<.02) features than clinicians.

#### Impressions of App Features

Diary and chart feature. Most parents and clinicians believed the diary and chart were crucial features for management of the child's asthma. One parent suggested improving the diary and chart features by adding a tagging feature to identify problem times of the year, where greater vigilance may help avoid an emergency room visit. One clinician felt that the existing diary feature does not assist with management and “the ideal tool would take kind of what you’re on, where you fall in your critical level, and give a re-, a recommendation.”

#### Self-Check Quiz Feature

Regarding the self-check quiz feature, one clinician said that “[k]ids are involved. They are more willing to be self-advocates for themselves.” One parent felt that the quiz was a helpful daily check to help her child better control his asthma. However, one parent said she didn’t know how valuable the feedback from the feature was and didn’t understand its purpose. Another parent felt it lacked long-term asthma monitoring capability because it didn’t save the results after showing the rating. One clinician stated that they would love to see the self-check quiz replaced with a validated measure of asthma control such as the Asthma Control Test and to also have the test result linked with clinical management steps for the patient, for example, “[i]f it’s low then you should call your doctor.”

#### My Allergies and Emergency Plan Feature

Parents stated that my allergies and emergency plan was a feature that would be helpful especially in places outside the home such as school. One parent stated the plan would allow her to “feel a little more at ease” knowing that her child would “have his emergency contacts right there in front of him.” In terms of communication, one parent liked that her child could show his basketball coach the plan so the coach would know what types of medicines he takes and when he takes them. One clinician brought up the benefits of using the feature for “engaging with them [patients]” including jointly inputting information into the plan and/or discussing what patients have previously input to ensure they are getting the correct guidance, especially regarding emergency situations.

#### Doctor Appointment Reminder Feature

Parents described the doctor appointment reminder feature as beneficial in helping them remember their child's medical appointments, which several parents said they sometimes forgot. The majority of clinicians saw it as crucial to ensure that their patients are staying on top of their asthma care. Multiple clinicians mentioned that appointment noncompliance is a problem, one stating that “[a]ny extra reminder that families have that they have an appointment I think is helpful.” However, the feature was also criticized by parents for its lack of integration into the app. One nurse offered a suggestion for improvement when she said,

If there was a way that mom can put it on parental controls and schedule the appointment or cancel the appointment and talk to our systems that will be a good thing as well too.

#### Medication Reminder Feature

Some parents saw value in the medication reminder feature in helping their child keep track of taking their asthma medicines. However, one parent stated that medication reminders would only be useful for adolescents who can't remember to take their medication. Some parents stated the medication reminder feature could be improved with an alarm. Most clinicians felt that the medication reminder feature would help their patients a lot, particularly with their controller medications, as they saw medication nonadherence as an important clinical issue.

#### Asthma Triggers Feature

Although one parent spoke positively about the trigger feature, this feature was more often criticized by parents and clinicians because of its lack of long-term monitoring and feedback capabilities. One clinician expressed the opinions of other participants when stating, “I don’t know what you’d [do] with it. Other than just be aware of it.” One parent suggested the asthma triggers feature be changed to provide trigger avoidance information when you click on each trigger. The inclusion of a global positioning system feature was also mentioned by one parent in order to track air quality by zip code. Related to communication, parents stated that the asthma triggers feature may prompt communication with the clinician and be beneficial in communicating a problem to others.

#### Doctor Report Feature

Parents expressed interest in being able to send updates to the doctor. Clinicians cited the feature as potentially good for monitoring their patients' asthma and determining next clinical steps. Related to communication, participants were generally supportive of the idea of using the doctor report feature as a way of collecting important clinical information, conveniently sending it to the doctor, and opening a communication channel. However, clinicians mentioned specific communication-related concerns including the Health Insurance Portability and Accountability Act (HIPAA) and security concerns, not wanting to be handed the report by families in clinic, or seeing the feature as impractical with difficulties getting it to interface well with office technology and to link to a patient chart.

#### Peak Flow Feature

Parents who did not find it useful said that they either did not use or rarely use a peak flow meter (a device used to measure how well air moves out of the lungs). Clinicians expressed some reservations to patient peak flow use, including worrying about the adolescent obtaining an accurate peak flow reading. One clinician would be interested in having patients blow into a device attached to the app to get an accurate peak flow.

#### School Form Feature

Some parents and clinicians felt that communicating asthma care between various entities using the school form feature would be very convenient. However, participants highlighted barriers to use including lack of applicability in which “not every school form is going to be the same” and “a lot of the schools want you to have–have the name of the school on it or the county on it.”

### Suggestions for New App Features or Components

One parent suggested adding additional reminder features to the app that would let their child know when to clean their spacers and when their medications need to be refilled. One doctor suggested pop-up messages tied to a medication log that would prompt patients with tips to improve medication use as well as setting the app to push automatic messages each day asking the patient about medication adherence and symptom levels.

In terms of education, parents stated that it would be helpful to have a frequently asked questions section, a visual library or location to display helpful tips, a tutorial showing the correct way to use an inhaler, and more specific information regarding asthma and medications, such as causes and treatments. Clinicians suggested including education for adolescents and their parents about asthma and asthma management, such as a section with pictures showing disease and medication information.

## Discussion

### Principal Findings

This study provides wide-ranging insight into how parents and clinicians perceive asthma app self-management features. We specifically explored how apps can involve these individuals who play key roles in the development and support of adolescents’ self-management skills [23], an endeavor few studies have undertaken [14-16]. With only 1 in 5 parents reporting ever having used an asthma app prior to this study, and to our knowledge, no app existing for adolescents that have both parent and clinician support features [12,13], we were able to gather valuable potential end-user feedback. Parents and clinicians in this study saw potential in using an asthma app related to three main themes: asthma monitoring and supervision, communication and information sharing, and education.

Although parents felt that their use of the apps for 1 week helped them better monitor their child's asthma, our findings that less than half believed the apps helped them better control their child’s asthma or follow doctors’ advice better were not surprising given that they were tasked with simply exploring the various feature of 2 existing apps. Further, neither of the 2 evaluated apps contained doctors’ advice or were equipped with specific components designed to actively involve parents or clinicians. While participants saw value in various features, such as the self-check quiz and asthma triggers, several critical comments revealed that the purpose of some of the features was not clear and several features lacked long-term asthma control and feedback capabilities.

Parents and clinicians provided generally positive feedback in terms of the apps potential for enabling communication and information sharing (eg, between clinician, parent, adolescent, and other social network members). This finding is important because research shows that adherence to treatment among adolescents with asthma is associated with adolescent’s perceived support (including self-management encouragement) from doctors [24]. Additionally, increased parental perception of increased parent-doctor communication has been linked to fewer pediatric office visits [25].

While participants saw the app as a device that simplified storing and tracking information as well as promoting data sharing, clinicians expressed some concerns about data transfer and security. Schneider et al [16] found that doctors perceived similar benefits (eg, a channel for accurate and credible information, a way to promote communication to engage adolescents in disease care) and barriers (eg, protection of personal medical information and security risks) related to using an asthma app as a way to connect with patients. Apps are often designed without enough or appropriate security and privacy measures in place to protect users’ health information [26]. There are numerous options for safeguarding health information and creating HIPAA compliant apps that allow for efficient and convenient data transfer [27] and these should be incorporated into new adolescent asthma app designs.

Regarding the theme of education, clinicians saw the app as a valuable forum through which to educate adolescents and a place to reinforce what was said in the doctor’s office. Parents talked in terms of the app as a teaching tool for the child, to enhance their own knowledge as well as a utility to provide education to others in the child's social circle. Collectively, these findings plus theme findings related to monitoring/supervision and communication/information sharing offer support for integrating information, decision support, and social support components, a needed methodological app template [11].

The knowledge that their child would have an app with an emergency action plan available (when needed) eased parental concerns. This finding concurs with another study showing parents believed their own anxieties would be relieved with better asthma control on the part of their children [15]. An app may be a useful device to promote treatment planning during office visits, as doctors rarely discuss action plans with children and their caregivers [28].

Interestingly, neither parents nor clinicians talked about the medication reminder feature in relation to them using the feature to assist the youth in any way, likely in-part, because the youth were teenagers rather than younger children. Findings that clinicians rated the medication reminder as the most useful feature are supported by another study showing that physicians believed medication reminders would assist adolescents in following their asthma action plan [16]. Parents ranked the reminder feature lower than clinicians, and in this example, where usefulness ratings differ among parents and clinicians, incorporating both views may lead to a design decision to allow users to hide the medication reminder feature, based on user preference. These and other study findings based on different stakeholder perspectives support research suggesting the need for a team approach involving parents and health professionals in the design, development, and evaluation of adolescent-centered asthma self-management apps [23]. Adolescents’ perspectives should also be considered in such design and development decisions [14,15,17,18,29-32]. Findings from our parent study show adolescents with asthma feel that asthma apps can positively influence their self-management behaviors and the majority of adolescents believed apps could enhance communication with their caregivers and medical providers [17,18].

Finally, parents and clinicians offered valuable suggestions for app improvements and new apps features. Participants thought that an app could be more useful if data entry was encouraged in a more consistent manner (eg, daily prompts encouraging specific input) and data output provided in a more useful and engaging way. Other studies have found that both parents and physicians think refill reminders would be helpful [15,16] and parents in one study embraced weather condition alerts that related to asthma trigger risk [15]. The need to ensure the use of validated measures was also suggested pointing to the need for evidence-based app content, something that is currently lacking in existing asthma apps [33,34]. Specific suggestions for new educational app components or features highlight valuable participant ideas that should be considered for incorporation into future adolescent asthma app designs.

### Limitations

The use of a convenience sample of parents and clinicians, the different interview modalities, the small sample size, and exploratory nature of this study limits our ability to generalize results to the larger population, especially to families and providers in rural areas who may have less access to cellphones [35]. Parents used the 2 asthma apps for a 1-week period and clinicians used them for 10-15 minutes, so they had real-world experience with the apps to inform their opinions about the usefulness and effects of various features. It is possible that giving both groups a longer period of time to use the apps would have yielded additional insights related to using the asthma apps. Several participants discussed having technical difficulties with various features, which limited their ability to provide detailed information in some cases. Also, although the 4 parents that did not complete the 1-week follow-up interview did not appear demographically different from the parents who completed the study, selection bias could have affected our results. Lastly, parent and clinician ratings of feature usefulness could have been inflated due to social desirability bias.

### Implications

An app that allows adolescents to optimally engage clinician and parent support could potentially improve adolescent self-management by allowing adolescents to obtain support in between doctors’ appointments [36]. The current study identified several features that may enhance and improve the ability of parents and clinicians to support adolescents as they self-manage their asthma. Incorporating components and features informed by the socioecological model or other frameworks that increase attention to social factors influencing adolescent’s engagement in asthma self-care (including two-way asthma care communication between families and clinicians and components involving families and clinicians that encourage adolescent self-management) will likely impact patient care and asthma outcomes, such as medication adherence and asthma control. Well-designed asthma apps have a great deal of potential to benefit adolescents with asthma and enhance asthma service–delivery processes as they are cost-effective, convenient, easy to access, and can be tailored to individual needs. More research is needed to compare and evaluate various aspects of intervention use and efficacy (eg, patient management, usability, acceptability, adoption, clinical outcomes) of newly designed apps.

### Conclusions

This study was conducted to inform the development of future asthma apps in order to engage important stakeholders who interact with adolescents to assist in asthma management and promote self-management skills. These findings provide valuable insight and revealed potential app and app feature requirements that were expressed as three main themes: monitoring/supervision, education, and communication/information sharing. Involving parents and clinicians in asthma app planning, development, and intervention activities is likely to result in more widely accepted, understood, and effective adolescent asthma self-management apps [23,36].

## Abbreviations

HIPAA: Health Insurance Portability and Accountability Act
RA: research assistant
